# Surrounding superficial esophageal cancer masked by *Candida* esophagitis that was difficult to distinguish from *Candida* esophagitis alone: A case report

**DOI:** 10.1002/deo2.70080

**Published:** 2025-02-14

**Authors:** Shunsuke Takahashi, Mitsuhiro Kono, Yasuhiro Fujiwara

**Affiliations:** ^1^ Department of Gastroenterology Osaka Metropolitan University Graduate School of Medicine Osaka Japan

**Keywords:** *Candida* esophagitis, endoscopic submucosal dissection, endoscopy, esophageal cancer, squamous cell carcinoma

## Abstract

During the initial diagnosis of superficial esophageal squamous cell carcinoma, a 70‐year‐old man was treated with endoscopic submucosal dissection (ESD). Two years after the first ESD, follow‐up endoscopy revealed that the extent of hyperkeratosis gradually expanded over the following 4 years; however, biopsies conducted only detected *Candida* mycelia and no cancer at that time and every 6 months for 4 years. Despite initiating fluconazole treatment for persistent *Candida* esophagitis 6 years after the first ESD, the lesions did not resolve, and the second ESD was performed 6 years after the first ESD, which revealed squamous cell carcinoma. This case highlights that esophageal cancer should be considered when localized, hyperkeratotic *Candida* esophagitis is unresponsive to antifungal treatments, especially with a history of esophageal cancer or high‐risk factors such as drinking alcohol and smoking.

## INTRODUCTION

The differential diagnosis for white flat esophageal elevations includes both esophageal cancers, such as verrucous carcinoma, and *Candida* esophagitis. The diagnostic process for esophageal cancer frequently involves a combination of endoscopy and histopathological examination. However, there are instances where a diagnosis cannot be made based solely on biopsy. This report presents the case of superficial esophageal cancer that could not be diagnosed solely based on biopsy findings.

## CASE REPORT

**FIGURE 1:**
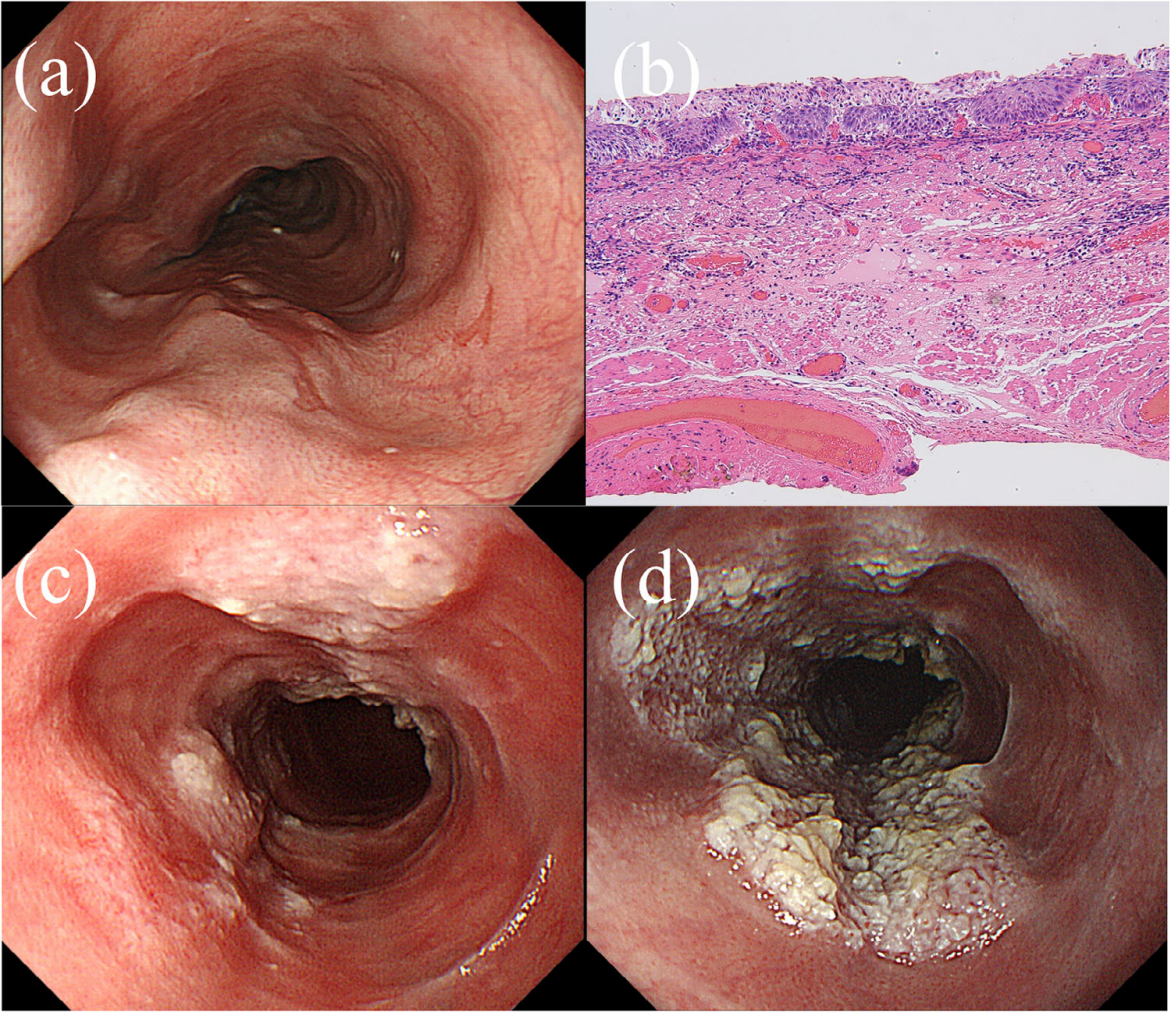
(a) Endoscopic image of the esophageal squamous cell carcinoma treated with the initial endoscopic submucosal dissection. (b) Histopathological examination of the resected lesion showed well‐differentiated squamous cell carcinoma in the epithelium (HE ×40). (c) A circumferential elevated lesion with hyperkeratosis was located in the mid‐thoracic esophagus during its initial detection. (d) Endoscopic image of the lesion 2 years after its initial detection.

**FIGURE 2:**
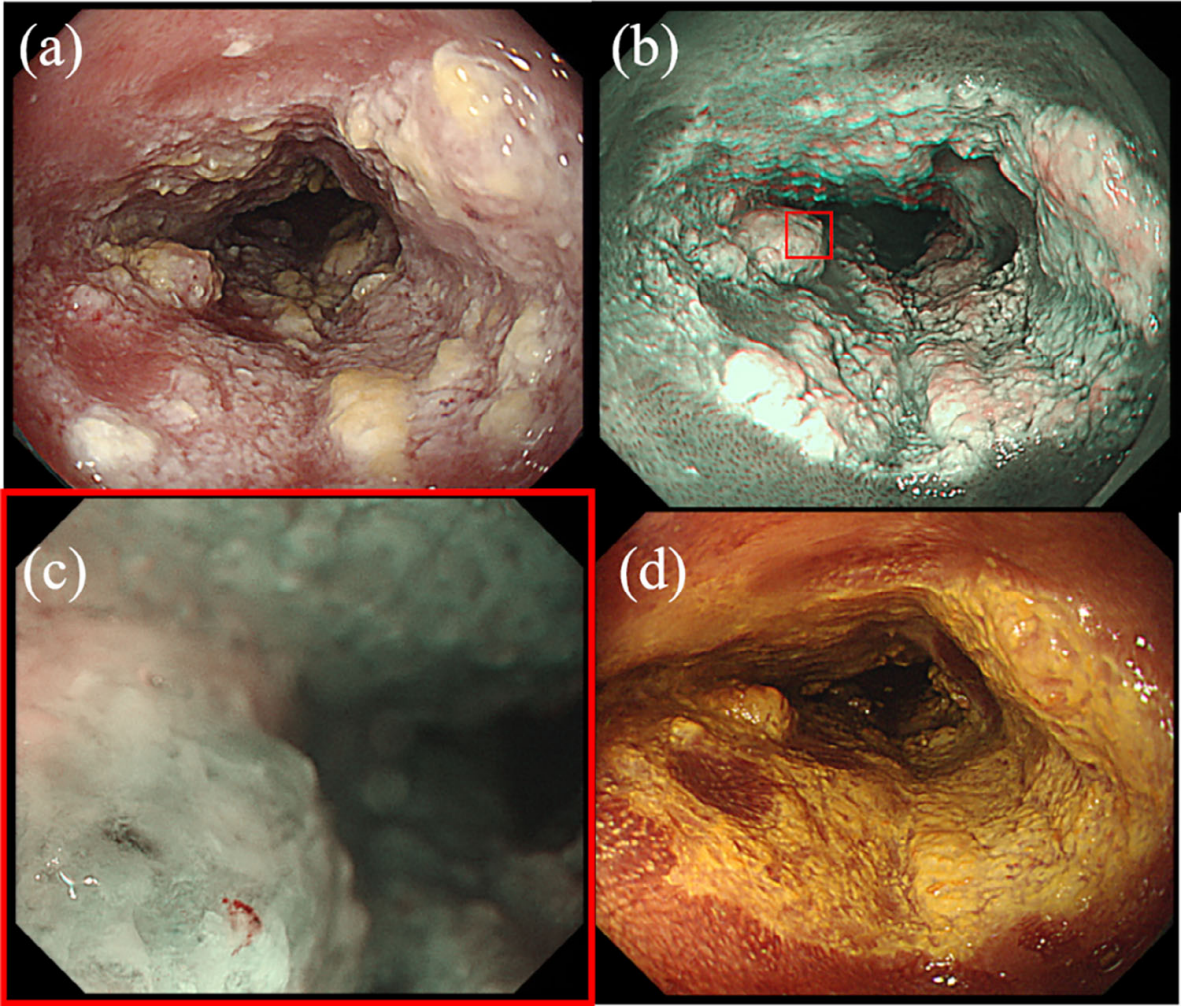
(a) Endoscopic image of the lesion before the second endoscopic submucosal dissection. (b) NBI showed hyperkeratosis and no internal vessel. (c) Magnified narrow‐band imaging showed no internal vessel. (d) Chromoendoscopy with Lugol staining showed an unstained area.

**FIGURE 3:**
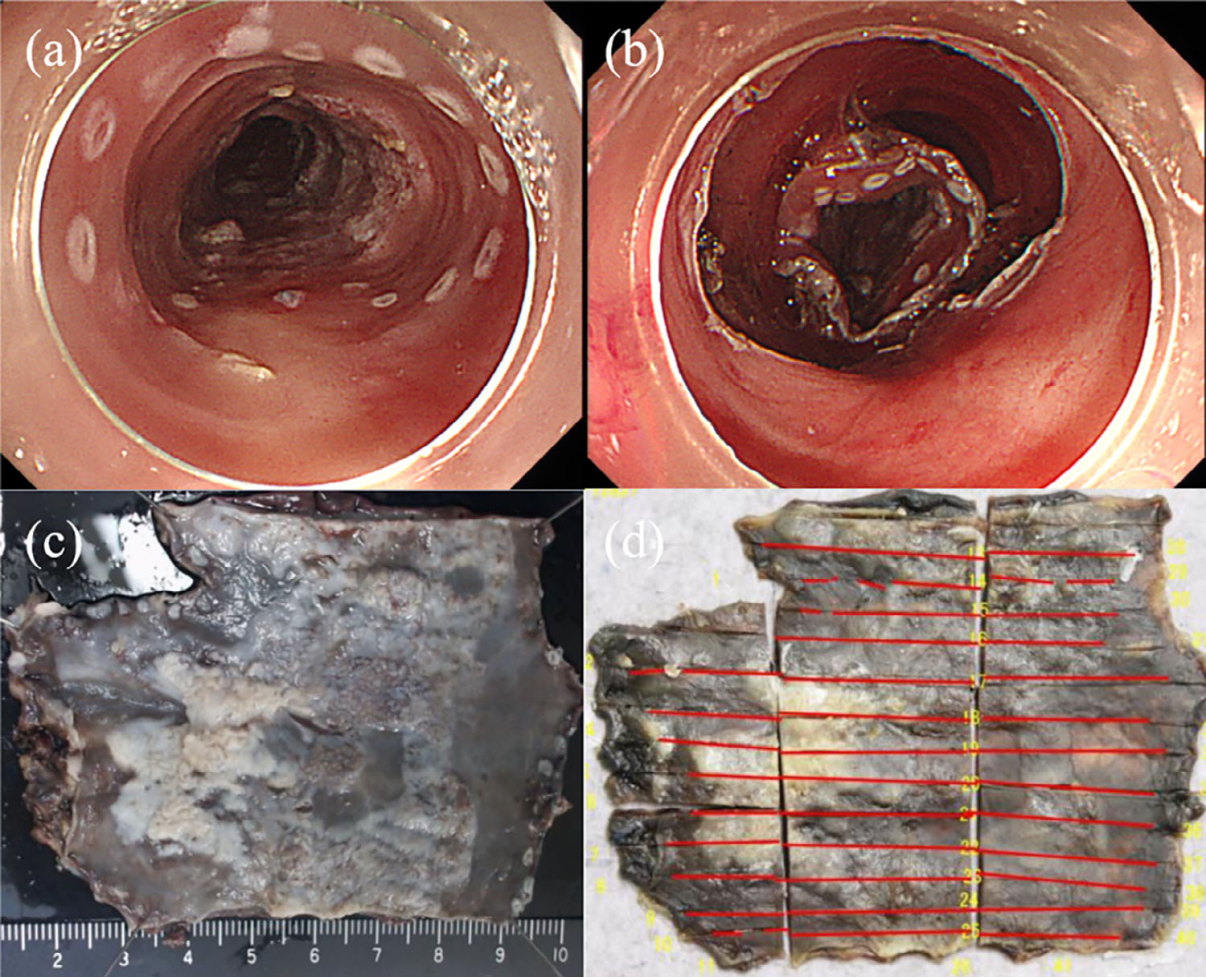
(a, b) Surrounding superficial esophageal cancer resected en bloc by endoscopic submucosal dissection. (c) The gross macroscopic image of the resected specimen. (d) Red lines map tumor cells.

**FIGURE 4:**
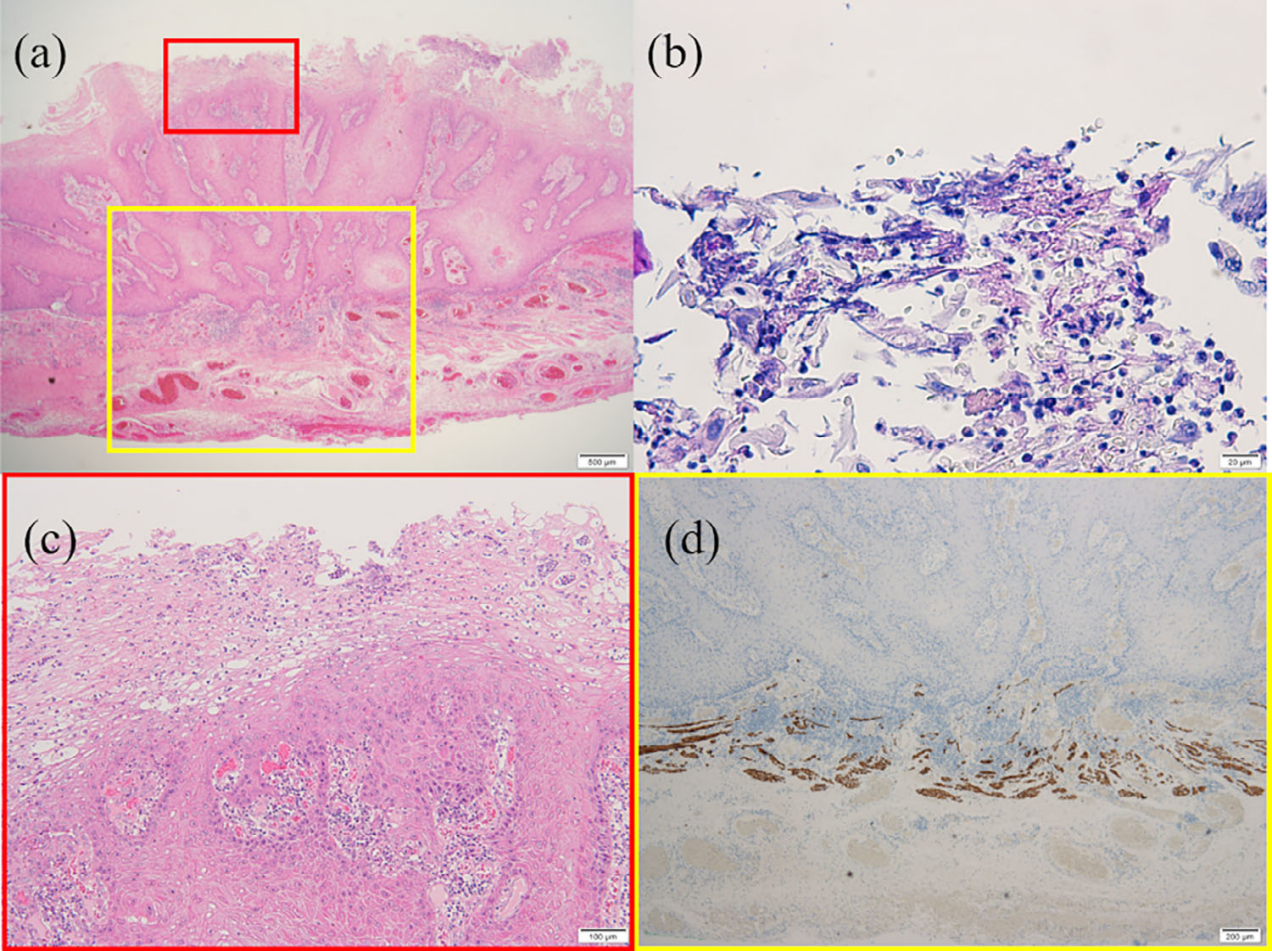
(a) A histopathological examination of the resected lesion showed squamous cell carcinoma and *Candida* proliferates at the epithelial layer (HE ×20). (b) Histopathological examination showed *Candida* proliferates at the epithelial layer (D‐PAS ×500). (c) The carcinoma showed infiltrative growth (HE ×100). (d) Histopathological examination of the resected lesion did not involve lympho‐vascular invasion (desmin ×250).

A 70‐year‐old man underwent endoscopic submucosal dissection (ESD) for superficial esophageal squamous cell carcinoma located 30–33 cm from the incisors (Figure 1a, b). The patient had a 47‐pack‐year smoking history (20 cigarettes per day) and has quit smoking completely. He consumed 20 g of alcohol daily for 40 years. The lesion was removed completely without any adverse events. Histopathological examination of the resected lesion showed well‐differentiated squamous cell carcinoma in the epithelium. After the first ESD, surveillance endoscopy was performed every 6 months. Two years after the first ESD, surveillance endoscopy revealed a circumferential, raised, slightly nodular esophageal lesion with hyperkeratosis at the mid‐esophagus, located 33–38 cm from the incisors (Figure 1c). The lesion demonstrated a gradual extension trend over the past 4 years (Figures 1c,d and 2a). Magnifying endoscopy with narrow‐band imaging revealed no significant vascularity on the surface of the lesion (Figure 2b,c). Chromoendoscopy with Lugol staining revealed an unstained area of the lesion (Figure 2d). Endoscopic biopsies were performed every 6 months for 4 years, and no cancer was detected during this time. However, *Candida* mycelia were detected on each pathological examination of the nodular lesions and the surrounding mucosa. The *Candida* fungus was found to be susceptible to fluconazole in a susceptibility test, and treatment with fluconazole was initiated. However, the lesions did not resolve after treatment with fluconazole. If this lesion were verrucous carcinoma of the esophagus, a histopathological diagnosis would be difficult using endoscopic biopsy specimens due to non‐specific inflammatory changes of superficial layers that tend to have less atypical epithelium. Given the patient's history of esophageal cancer, high‐risk factors such as drinking alcohol and smoking, and most importantly, the ineffectiveness of antifungal treatment, an ESD was performed for diagnostic and therapeutic purposes after obtaining informed consent from the patient. Thus, a second ESD was performed 6 years after the first ESD for diagnosis (Figure 3a–d). Histopathological examination revealed *Candida* proliferates at the epithelial layer, epithelial parakeratosis, and well‐differentiated squamous cell carcinoma in the epithelium and lamina propria mucosae, consistent with a raised lesion (pT1aN0M0 pStage1A; Figure 4a,b). The carcinoma showed infiltrative growth (Figure 4c). Therefore, the patient was diagnosed with well‐differentiated squamous cell carcinoma underneath *Candida* esophagitis. The squamous cell carcinoma did not extend beyond the lamina propria mucosae, had clear horizontal and vertical margins, and did not involve lympho‐vascular invasion (Figure 4d). Verrucous carcinoma is characterized by a highly differentiated histological appearance and a well‐preserved basal membrane of the epithelium. In this case, although there was a relative lack of atypia and notable hyperkeratosis, papillary growth was not clear, and the pattern of invasion in the infiltrative part resembled that of typical infiltrative growth rather than verrucous carcinoma. The patient has had no recurrence for 2 years following the second ESD.

## DISCUSSION

The differential diagnosis for diseases causing white flat elevations in the esophagus includes superficial esophageal cancer, *Candida* esophagitis, leukoplakia, or hyperkeratosis.^1^, ^2^ Superficial esophageal cancer complicated by *Candida* esophagitis may not always be detected through endoscopy. This case was of a circumferential superficial esophageal cancer, followed over a long period, complicated by *Candida* esophagitis. Verrucous carcinoma is characterized by a highly differentiated histological appearance and a well‐preserved basal membrane of the epithelium. In this case, although there is a relative lack of cellular atypia and notable hyperkeratosis, the papillary development is unclear. The pattern of invasion in the infiltrative part resembles typical infiltrative growth rather than that of verrucous carcinoma. As Candida esophagitis progresses, the lesions frequently merge and elongate, with severity determined based on the size and number of white plaques, ulcer formation, and edema.^3^ *Candida* esophagitis can be difficult to distinguish from tumors through endoscopic observation, especially when there is severe edema and mucosal changes. In this case, in addition to the typical white plaques, the presence of surrounding flat elevations led to the consideration that there might be tumors underlying the *Candida* esophagitis. Furthermore, the clinical manifestations of the patients are often related to the extent of esophageal mucosal damage.^4^ Therefore, the lack of common symptoms of severe *Candida* esophagitis, including pain with swallowing, difficulty swallowing, and pain behind the sternum, also serves as an important clue in considering the possibility of other diagnoses.

Chronic *Candida* esophagitis could be a risk factor for esophageal cancer.^5^, ^6^ In this case, esophageal cancer may have been present but hidden underneath the *Candida* esophagitis. Retrospective analysis of biopsy specimens collected during the surveillance period revealed no findings suggestive of esophageal cancer. However, the presence of *Candida* esophagitis, along with hyperkeratosis and epithelial thickening, may have masked accurate sampling of the cancerous area, making detection challenging. Alternatively, long‐standing inflammation caused by chronic *Candida* esophagitis may have promoted the development of esophageal cancer, as the extent of hyperkeratosis gradually expanded over the past 4 years, which likely reflected disease progression. Although cancer was not detected every 6 months for 4 years, serial endoscopic surveillance revealed a progressive extension of the lesion over time, indicating that ongoing inflammation may have modified the esophageal mucosa, leading to the development of cancer.

In conclusion, this case highlights the importance of considering both the masking effect and the carcinogenic potential of persistent *Candida* esophagitis, especially in patients with a history of esophageal cancer or high‐risk factors such as drinking alcohol and smoking. It is necessary to rule out esophageal cancer in cases where there are multiple occurrences of white flat elevations resembling *Candida* esophagitis. Diagnosis should not be based solely on a single biopsy. A multifaceted examination approach is required, and at times, diagnostic treatments such as ESD are necessary.

## CONFLICT OF INTEREST STATEMENT

None.

## ETHICS STATEMENT

Approval of the research protocol by an Institutional Reviewer Board: N/A.

## PATIENT CONSENT STATEMENT

Informed consent was obtained from the patient.

## CLINICAL TRIAL REGISTRATION

N/A.
